# Comprehensive Approaches to Improving Nutrition: Future Prospects

**DOI:** 10.3390/nu11081760

**Published:** 2019-07-31

**Authors:** Syed M. Shahid, Karen S. Bishop

**Affiliations:** 1 Faculty of Healthcare, Aspire2 International, 1023 Auckland, New Zealand; 2 KIBGE, University of Karachi, 75270 Karachi, Pakistan; 3 Discipline of Nutrition and Dietetics, School of Medical Sciences, FMHS, University of Auckland, 1023 Auckland, New Zealand; 4 Cancer Society Research Centre, School of Medical Sciences, FMHS, University of Auckland, 1023 Auckland, New Zealand

**Keywords:** community, government, implementation, malnutrition, obesity, over nutrition, schools, sugar tax, regulation

## Abstract

When it comes to nutrition, nearly everyone has an opinion. In the past, nutrition was considered to be an individual’s responsibility, however, more recently governments have been expected (by some) to share that responsibility by helping to ensure that marketing is responsible, and that food chains offer healthy meal choices in addition to their standard fare, for example. In some countries, governments have gone as far as to remove tax from unprocessed foods or to introduce taxes, such as that imposed on sugary soft drinks in the UK, Mexico, France and Norway. Following on from the sugar tax, chocolate might be next! Is this the answer to our burgeoning calorie intake and increasing poor nutritional status, or is there another approach? In this narrative we will focus on some of the approaches taken by communities and governments to address excess calorie intake and improve nutritional status, as well as some of the conflicts of interest and challenges faced with implementation. It is clear that in order to achieve meaningful change in the quality of nutritional intake and to reduce the long-term prevalence of obesity, a comprehensive approach is required wherein governments and communities work in genuine partnership. To take no or little action will doom much of today’s youth to a poor quality of life in later years, and a shorter life expectancy than their grandparents.

## 1. Introduction

Despite advances in food science and technology, and sufficient food production to feed the global population, malnutrition remains prevalent. In addition to malnutrition, obesity is rising in low, middle- and high-income countries and surprisingly, often sits alongside malnutrition in low income communities [1]. Nutrition impacts the incidence of chronic diseases and can enhance the quality of the years gained through advances in modern medicine and safety practices. Obesity has reached epidemic proportions in many countries (Canada, USA, Mexico, the United Kingdom, the United Arab Emirates, Australia and New Zealand, amongst others), and is a serious threat to health and life expectancy. It is well recognized that behavior and attitudinal change is difficult, yet essential to achieve in order to optimize nutrition [2]. In many countries, nutrition is seen as an individual responsibility, but as a greater understanding is gained of the power of marketing and the food environment, so we are recognizing the role governments could, and in some cases, do play in influencing nutritional intake. In this article we define community as “the condition of sharing or having certain attitudes and interests in common” [3] as all groups mentioned herein are involved in improving nutrition and reducing excessive calorie intake in various groups of people, or in the national or global population as a whole. In general, we refer to the national community, of which governments form an important part.

In addition to international agencies and national governments, activists and local/national community groups have a role to play in bringing about change. Is it purely about lobbying governments to regulate the content of foods, to ensure responsible marketing, and educate the populace regarding calorie content and nutritional value? Or could these groups also work within their communities to bring about improved nutrition? What might this “work” look like?

In this article we will discuss how governments and community groups have implemented various approaches in an effort to bring about change to improve nutrient intake and/or reduce calorie intake. The critical role of nutrition in health and well-being, learning, and efficacy of people, and its relationship with economic development, is well-established, and findings have been published by various researchers [4,5,6], and therefore will not be discussed herein. 

In this article we will provide examples of interventions that have been successfully used to improve nutrition and suggest ways in which obesity could be addressed through a comprehensive broad community approach and thus improve the future prospects of today’s youth. As far as recent and relevant research work carried out by nutrition and health institutes and authorities are concerned, the work to be done includes nutrition policies, preparation of appropriate supportive regulation, attention to vulnerable regions and populations, and regulation and control of the price of food products. However, it is possible for price control to have a negative impact on low-income communities [7], hence the necessity for continual data collection and evaluation. Regulations, policies, guidelines and price control are not the only ingredients for nutritional change, and education, counselling and other means of support are also required.

In this narrative we will discuss how governments have influenced food consumption with respect to food security, malnutrition, poor quality diets and excess calorie intake. In addition, we will discuss government-community partnerships, and the outcome of relevant intervention studies to reduce calorie intake and improve the nutritional quality of dietary intake.

## 2. Nutritional Policies and Action Plans — A Government Perspective

There have been a large number of studies and reports wherein goals and objectives are set in order to eliminate and/or reduce food and nutrition insecurity, as well as to reduce different forms of malnutrition [8,9,10]. Despite all these efforts, the advancement to achieve the set goals and to meet the objectives has been found deficient compared to the intentions. This displays a clear continuation of existing trends which will leave millions of people under/malnourished. At the same time, malnutrition early in the life cycle presents a danger to negatively impact upon health and performance later in life, and for malnutrition in one generation to negatively impact the next, and thus manifestations may be felt far into the future [11].

In the proceedings from a series of food-related conferences, particularly the Global Food Summit (2019), World Food Summit (2019) and New Zealand (NZ) Food Summit (2017), it was observed that as early as the 1990s, many countries had developed national nutrition plans and policies in the following major action and strategic areas:

- Conventional nutrition targets to be set as per programs and policies;
- Household food to be improved with subsequent nutrition security;
- consumers to be protected via improved food quality and safety,
- Breastfeeding to be promoted and increased;
- Socioeconomically deprived and nutritionally vulnerable populations to receive improved support;
- Specific micronutrient deficiencies to be prevented and controlled;
- Healthy dietary patterns and lifestyle to be promoted;
- The above-mentioned major action and strategic areas need to be assessed, analyzed and monitored.

The priority that governments are placing on nutrition is well-evidenced, as 149 (78%) World Health Organisation (WHO) Member States had begun to put into place their nutrition related commitments made in 2003, while another 17 (9%) had plans and policies under preparation [12]. Through this initiative, comprehensive technical and financial support has been provided by the WHO to governments that are actively developing and implementing national policies and programs that are likely to improve population nutrition, and thereby improve health outcomes. A concerted effort is being made by the WHO to support nations to address food and nutrition related problems at a local level [13], and for some countries, the WHO documents/reports form an integral part of their nutritional interventions (e.g., NZ [14]).

### 2.1. Government Roles — What Needs to Be Done? 

Traditionally, agriculture, nutrition and health sectors have operated as separate entities, and therefore policies and government structures have been designed without looking closely at the interactions among these sectors. Clearly these sectors are inter-related and should not be considered in isolation. Nonetheless, we have considered nutrition herein because of the impact on health.

National governments’ have the opportunity to perform a number of important functions with respect to generating and disseminating information about the relationship between diet and health. One such role includes the funding of research to support the generation of basic scientific knowledge. Governments may also play a role in education and in shaping the types of information available to consumers. Among the issues highlighted by Ippolito (1999) are the importance of incentives in determining the types of products offered for sale, the key role of scientific uncertainty and the dynamic nature of diet–health knowledge in shaping regulatory choices, and finally, the importance of recognizing consumer heterogeneity in assessing the success of regulatory rules and other government initiatives [15].

The nutritional well-being and health of a population is an indicator of national development, and as such reflects the combined performance of social, economic, agriculture and health sectors [16]. Nutrition is also an essential ingredient for national development, with a healthy, well-nourished and educated population being the ideal foundation for promoting national economic growth. Development aims to provide all people with the social and economic environment necessary for them to lead active, healthy lives. For achieving this objective, development policies and programs need to be directed toward improving human development, including improving nutritional well-being.

Potentially, governments have the power to apply a broad spectrum of plans and policies that include voluntary and mandatory. This strategy may include, but is not limited to, a bill (proposed law), statute/act/law which is approved by executive and legislative bodies, agency implementation (interpretation, application, regulation), court decision, guideline (recommendation, not mandatory), or directive (internal to an institution). 

Public health concerns such as nutrition are multifactorial by nature. Multidimensional effects can be induced by very simple or even single interventions within rather complex interactions [17]. Plans and policies can be made that are spot targeted to the food and nutrition problems rather than more indirect mechanisms related to marketing, retailing, trading, farming, food processing, general education followed by regulations, and economic authorization. Each strategic intervention can be itemized as per specifically related characteristics which are to be measured and defined in planning and policy design (Huang et al., 2018). However, it is important to consider if these spot targets are likely to be successful in isolation.

For the different government policy actions, the following points may be presented as per their SWOT (Strength, Weakness, Opportunity, Threat) analysis:

- Appropriate education for the population in general;
- Labelling regulations for point of purchase;
- Taxation incentives and otherwise (Sugar tax, Cigarette tax, Chocolate tax, Alcohol tax etc.);
- Assistance for the supply of food for the needful;
- Setting and maintenance of nutritional standards;
- Setting up and regulation of quality standards;
- Food marketing standards;
- Collaborative research, development and innovation;
- Coordination of actions across ministries, agencies, and at local, national, and international levels [18].

### 2.2. How Policies Can Change the Food System

A number of lifestyle indicators have been associated with rising income and urbanization. Availability of/and variety of food, price and social/cultural acceptance greatly affect the eating habits of a particular population (HLPE, 2017). People’s dietary selections and their nutritional status are greatly influenced by their food system and sub-systems. The food systems and sub-systems actually engage the consumers to make specific decisions about obtaining, preparing and consuming food (FAO, 2004).

The current nutrition situation may also be driven by the food systems currently available in a specific environment, which often does not provide the appropriate food needed for optimum health and wellbeing. The availability and affordability of healthy food has been challenging, and therefore it is important to improve food environments especially for vulnerable groups. The food system and sub-system’s role in driving the food related crisis, such as obesity, may not yet be fully appreciated, especially in countries that have, until recently, been struggling to combat hunger and under-nutrition. At present, a large number of policymakers from the health sector may have a limited understanding of the nature and magnitude of the problems posed by non-communicable diseases (NCDs), overweight status and obesity, which requires serious action on the part of everyone if these problems are to be prevented and controlled. 

Legislators are well placed to guide and monitor public sector policies and budget allocations. The right incentives can also be encouraged to support the adoption, and disincentives for action, by consumers and businesses, to transform the food system in order to deliver healthy food. Depending on the individual country context, various policy and legislative measures can be taken within different sub-systems of a food system. A number of food sub-systems can be taken into consideration including, but not limited to, agriculture food production, food storage and transportation to food transformation, food retailing and provisioning etc., some specific examples are provided herein.

#### 2.2.1. Sugar Tax 

With sugar linked to a host of public health problems such as burgeoning obesity rates, type two diabetes (T2D) and dental carries, amongst others, a growing number of governmental bodies are initiating plans to reduce sugar intake. The year 2016 has been referred to as "the year of (the) sugar tax”, triggered by nutrition advocacy from social media influencers, celebrity chefs, and health and political groups as well as the publication of a WHO report (2015), which detailed the negative effects of sugar on health [19]. Over 35 national governments, states and cities now have sugar taxes (e.g., France, Mexico, Portugal, Saudi Arabia and the United Arab Emirates); 20 of which have been introduced since 2015 [19]. All this is a bid to reduce the incidence of T2D and obesity. Some locations (e.g., Hungary) have introduced “health taxes” beyond sugar-sweetened beverages (SSB), such as taxes on products with excess sodium, saturated fat and trans-fats.

Areas with sugar taxes already in place may be more likely to extend taxation to additional food and beverage categories. Health taxes have raised significant funds with little public opposition, although such taxes do not always raise funds as the cost of implementation and monitoring can be significant. Industry and political opposition has had a mixed impact with only a few countries (e.g., Denmark) fully abandoning taxation efforts. The impact can depend on the strength of the opposition, how closely industry and politics are intertwined, and the expected versus actual outcome/s from the health tax. In response to current and pending taxes, many global brands have committed to significant nutritional optimization, likely in response to social and governmental pressure. Reformulated products can deliver better nutritional profiles but maintaining taste is a challenge. The launch of low-, reduced- and no-added-sugar products has been muted, but steady [19].

In contrast, Denmark and Iceland have removed the taxes they previously placed on unhealthy foods. In response to the tax in Denmark, people shifted to purchasing cheaper products and purchasing goods in neighboring countries that do not have an equivalent tax [20]. Nonetheless, consumption of saturated fats decreased by 10-15% [20], and the removal of the tax was thought to be political rather than due to a lack of health benefit [21].

A sin tax can sometimes have a negative impact. In France the rise in SSB increased by more than the tax, likewise in Mexico [22,23]. In Mexico, the introduction of an SSB tax only decreased consumption of SSB by 6%, although the decrease in consumption has increased the longer the tax implementation [24]. Additional follow-up is required as this was an observational study capturing data from an unbalanced panel of households during the first two years of SSB taxation. People on low incomes spend a larger percentage of their income on SSB and other unhealthy foods, than wealthy people, and although the consensus seems to be that people on low incomes tend to be unresponsive to the price of SSB [25], those on low incomes in Mexico decreased their purchase of SSBs more than people on higher incomes [24].

#### 2.2.2. Salt Tax

Excess dietary salt intake is associated with elevated blood pressure, a major risk factor for cardiovascular diseases [26]. Salt reduction has been described by the WHO as one of the best investments to improve public health and an efficient and cost-effective way to decrease the burden of elevated blood pressure and cardiovascular diseases [27]

In 2013, WHO Member States adopted the global target of a 30% reduction of mean population intake of salt by 2025, as part of a broader set of strategies to reduce premature mortality from non-communicable diseases by 25% in 2025. A growing number of countries are developing and implementing strategies to reduce salt intake, including, but not limited to, food supply reformulations, front of package labelling, taxation, consumer education, and interventions in public institutions. However, in many countries, these strategies are voluntary or restricted to a limited number of food products [28].

The Republic of South Africa was the first country globally to develop comprehensive, mandatory legislation to reduce sodium levels across a wide range of processed food categories, which involved the co-operation of many food industry members from various sectors [29]. It is estimated that about half of the daily salt intake in South Africa derives from processed foods, with bread being the greatest contributor to non-discretionary salt intake. The South African sodium legislation was passed by the Department of Health in 2013 and set restrictions regarding the maximum levels of sodium allowed in several commonly consumed foods which, in addition to bread, includes breakfast cereals, margarines, meat products, snack foods, and soup mixes [29]. In the year leading up to the mandatory implementation date, approximately two-thirds of foods that fell within the legislation met the salt requirements, whilst nearly 50% of those that did not, were within 25% of the target [29]. Clearly the legislation is having an impact.

#### 2.2.3. Tobacco Tax

Although not a food, tobacco use provides us with a good example of how governments may be conflicted, introduce legislation to modify behavior and improve health outcomes. Such policies and interventions may include marketing, packaging, warning labels, taxes, education and smoking cessation programs.

In New Zealand and numerous other countries, cigarettes and tobacco products have provided a reliable source of revenue to successive governments for many years, as tobacco is very heavily taxed. The government will go ahead with a 10% hike in the tobacco excise in 2019 but has not decided on a further rise in 2020. This is despite a report saying price increases force some families to decide between food and other essentials, or their tobacco fix [30]. Although it is agreed that the purpose of the tax is to discourage people from using tobacco, there is a dispute as to whether taxation in this situation is beneficial or harmful. In other countries, governments have an additional vested interest in the continuation of tobacco use when tobacco companies are fully or partially state owned e.g., China and Russia.

The WHO 2009 report on the global tobacco epidemic noted, “while more data and analysis are needed on tobacco’s costs and economic burden, it is clear that its economic impact on productivity and health care—already disproportionately felt by the poor—will worsen as tobacco use increases” [31]. Indeed, in some areas of the world, women and men use other tobacco products more than they use cigarettes. For example, in India, 12% of women chew tobacco, whereas only 2.4% of women smoke manufactured cigarettes. As many economic concepts, policies, and practices are relevant to a gender analysis, they are presented here to identify gaps needing to be addressed in further research [32].

Similarly, at the household level, in Indonesia, where smoking is most common among the poor, 15% of total expenditure of those in the lowest income group is on tobacco, while the poorest 20% of households in Mexico spend nearly 11% of their income on tobacco [33].

We are of the opinion, that in the long-term, sin taxes are likely to have the desired effect of influencing consumer behavior, particularly if off-set by removal of taxes or subsidies on unprocessed nutritious foods, however, indirect taxes (removal or addition) are only part of the complex approach required to achieve a global or community shift in dietary practices.

## 3. School-Based Approaches with and without Community Involvement

Schools are one of the easier places to implement an intervention, as there is a captive audience that is not independent and therefore ample opportunity to encourage healthy eating and appropriate levels of physical activity, and facilities are available for physical activity [34]. It is therefore not surprising that many such interventions have been carried out in schools. However, it is still possible and beneficial for communities to be involved in school-based interventions. School-based approaches to improving nutrition, reducing calorie intake and thus reducing the incidence of obesity can take a number of forms. Programs can take the form of education of students, or students and parents/families, interventions to bring about behavior change, a change in the school nutritional environment, as well as involvement of families and the community, and a combination of some or all of these approaches.

It has long been recognized that community groups are part of the community they reside in, and therefore are well placed to initiate and support change in their communities. Hoelscher et al. showed that a school-based intervention (Coordinated Approach To Child Health — CATCH) to decrease the prevalence of children who were obese or overweight, showed a greater impact when the community was involved, rather than when they were not [35]. The integration of the community into the CATCH program included a community member on the CATCH committee, “Best Practices” workshops for the CATCH community and the promotion of an activity guide to support the school program and community.

The Active for Life Year 5 intervention, whereby teachers received training and materials to provide a lesson and an interactive homework plan, and parents were provided with guidance in a school newsletter, showed no effect on fruit and vegetable intake or weight loss [36]. An evaluation of the lesson, teacher attitude, assessment of whether the interactive homework was carried out, or whether the parents read the relevant section of the newsletter and how it was perceived, was not carried out. Education on its own is insufficient to effect change. If we wish children to stop consuming certain foods, then why are we supplying many of them through school cafeterias and vendors?

Although a number of successful interventions have been published, these interventions have not been widely implemented, for example, across the globe schools have failed to implement government policies to decrease the availability of unhealthy foods in schools [37,38,39]. Damschroder et al. (2009) suggest that interventions that are complex, expensive, require skills that are not commonly found in schools, or are time consuming, are unlikely to be adopted [40], and this may explain this lack of implementation. Wolfenden et al. implemented a randomised controlled trial (RCT) to test a strategy to improve the implementation of a policy to enhance the quality of foods available in primary schools in New South Wales, Australia [41]. The strategy was effective in reducing total fat intake, but not the number of calories (Table 1). 

A number of interventions targeted school lunches and/or breakfasts as this is both practical, measurable and is likely to impact a large percentage of school children. Implementation included reduced sodium-levels, reduced total fat content, increased fruit intake and increased calcium intake, amongst others [41,42,43]. Table 1 and Table 2 show the mixed results with respect to dietary and anthropometric outcomes respectively, from nutritional intervention studies carried out in schools.

Although some strategies for school-based interventions have been established and proven to be successful, the correct implementation of these strategies is lacking. With respect to the implementation of government policies in schools, many difficulties were experienced. In the USA there is an annual bidding process whereby food items are selected a year in advance and the order needs to closely match projected student purchasing if waste is to be avoided [43]. In addition, schools can profit from student purchases, and therefore they wish to sell what the students will readily purchase [43]. In South Africa time and purchasing power were held partially to blame for the lack of implementation of nutritional interventions in a low-income setting [46], whilst in the School Nutrition Advances Kid (SNAK) study, one limitation included the low response rate of students completing the dietary survey, due to the lack of signed consent forms [45].

Based on the Cochrane Review carried out by Wolfenden et al. [59], to investigate “strategies to enhance school-based policies or practices targeting risk factors for chronic disease” it was clear that the quality of the data around interventions was low and therefore conclusions could not be drawn. In this review by Wolfenden, wherein 27 relevant trials were assessed, it was unclear whether implementation of strategies influenced staff knowledge or attitudes, whether it influenced outcome with respect to student health behavior or was cost-effective [59].

Data are conflicting, with many studies showing benefit, particularly with increasing healthy eating habits, and fruit and vegetable intake [60,61,62,63], whilst numerous other school based approaches have been shown to be ineffective and it has been suggested that a broader more comprehensive approach might be necessary [64,65]. A more comprehensive approach would avoid the situation that Whatley Blum et al. (2011) experienced when “banned” foods (as per the guidelines for schools in the USA) were removed from the cafeteria, yet similar sugar-sweetened products remained and advertising of the banned products was prevalent in the schools [49]. Similarly, de Villiers et al. (2015) felt there was insufficient buy-in from the parents/caregivers and teachers, although they did acknowledge that poverty was also a contributing factor to the failure of the intervention [46].

In the USA, the National Association of State Boards of Education (NASBE) proposed nutrition standards for consultation with the public in order to establish standards for foods sold in schools (other than those provided during the breakfast and lunch programs). These standards were consistent with the Dietary Guidelines for Americans and provided details regarding the type and percentage fat, sodium, caffeine, sugar and calories foods could contain per portion. Compliance with the guidelines is the responsibility of the school, with monitoring of compliance being administered by state education agencies http://www.nasbe.org/education-issue/student-health/.

**Figure 1:**
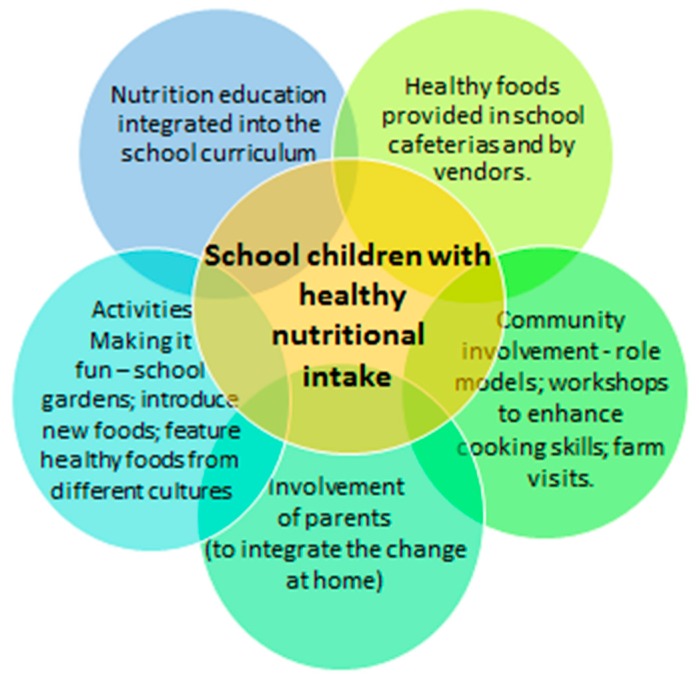
An integrated and comprehensive school-based approach to improve the quality of nutrition and decrease the calorie intake amongst school children (adapted from two Shaping Healthy Choices Programme webpages [44,48]).

Comprehensive school-based interventions include nutrition education (classroom curriculum) and promotion, family, community, school food environment, link to regional agriculture and a school wellness policy https://cns.ucdavis.edu/programs/shcp (Figure 1).

## 4. Government-Community Partnership Approaches

Comprehensive approaches involving researchers, schools, local and national authorities, and local communities are usually essential for an intervention where the outcome is behavior change [46]. A few examples will be presented where such approaches were utilized.

**Figure 2:**
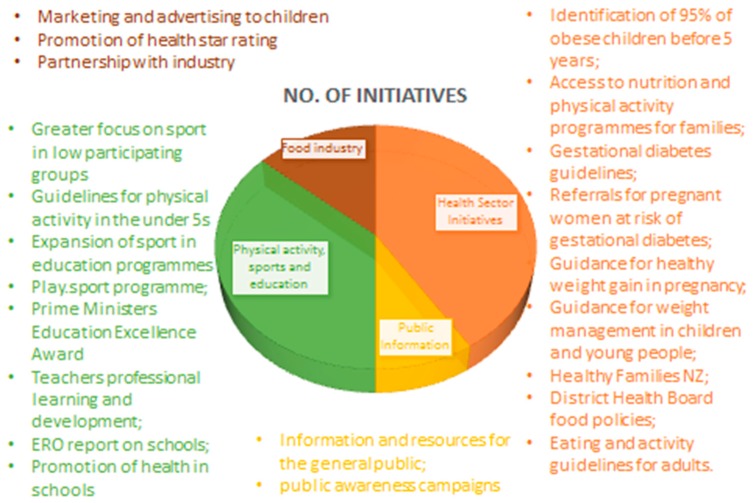
The initiatives outlined in the New Zealand Childhood Obesity Plan. Initiatives can be broadly categorized into government agency led, physical activity focused, health sector focused, or involving the food industry. Adapted from a New Zealand Ministry of Health report [14]. ERO-Education Review Office.

NZ launched a “Childhood obesity plan” in October 2015 with the introduction or expansion of 22 initiatives to prevent and manage obesity in those up to 18 years old (Figure 2.) [14]. The initiatives focus on foods and physical activity and involve communities, schools, families, the private sector and government agencies i.e. the plan is comprehensive and draws on evidence from the WHO Commission on Ending Childhood Obesity, as well as recent evidence from NZ and guidance from a technical advisory group. A baseline report was published in 2017 with clear points of measurement linked with childhood obesity indicators [14].

Part of this childhood obesity plan is a Green Prescription (GRx) to promote active families. This is a community-based approach supported by a government program. Referrals often come from GPs and the program involves physical activity and healthy eating advice, setting health related goals, cooking demonstrations, how to read food labels, education sessions, face to face meetings and home visits [14]. From a survey carried out in 2018, 43% of respondents receiving a GRx lost weight, and 61% are more active than they were prior to receiving a GRx [66]. Obese and overweight children are also enrolled on the program, and once they have graduated from the program lifestyle changes are supported by linking to community activities [14]. We see this ongoing support, at a community level, of lifestyle changes related to nutrition and physical activity, as being essential to the long-term integration of these changes.

In Scotland, an organization named Community Food and Health (CFHS), supported by the National Health Service, Scotland, work with and within low income communities to support people to access healthy and affordable food [67]. The community is also involved in research to determine cooking skills, ethnic attitudes to obesity and healthy foods, amongst other topics. Newsletters, blogs and other resources are made available to support healthy nutrition from infants to the elderly [67].

Another example is the Peterborough Environment City Trust PECT, which is a charitable organisation with a vision for sustainable places. It works to support children and families to adopt healthy behaviors by improving understanding, offering training to teachers as well as lesson plans, assistance with implementing vegetable gardens in schools, as well as family friendly activities to increase physical activity and people’s connection to food and nature [68]. PECT partner with “Everyone Health”, which is a broader network in the Cambridge and Peterborough areas in England to improve health and wellbeing in children and young people and they are supported by local councils. 

In India, a number of community managed initiatives have targeted malnutrition [69]. Several of these initiatives have successfully utilized funding from the World Bank, local government subsidies, and activities within the local community to cover the costs of the programs. Similarly, structured programs could be applied to over-nutrition and poor nutrition, thereby creating community environments that are conducive to healthy eating.

## 5. Future Prospects

In order to reduce the prevalence of obesity and improve population health, we need to improve the quality of nutrition and reduce the number of excess calories people consume. This is a challenging task and requires the co-operation of the wider community such that multiple organizations initiate and maintain a broad and comprehensive approach to the problem [13]. Without a comprehensive approach to poor nutrition and obesity, the future looks bleak, with crippling health care costs, and today’s youth experiencing reduced quality of life due to NCDs and shorter life expectancies than their grandparents.

No longer is our primary global concern about food security, but rather the quality of foods and the obesogenic food environment. Expansion of cities has changed dietary habits and patterns, and diets now contain more carbohydrate and fat-rich components with limited or no fiber. Although it might not be possible to change migration to the cities, it is possible to change the food environment with concerted and coordinated effort.

Implementation of global and national policy actions must be accompanied by systematic surveillance and evaluation to assess progress and guide further efforts. To date nutritional policies have failed to meet the nutritional challenges facing low, middle- and high-income countries. The epidemic of obesity is rapidly gaining momentum. In Iran, the incidence of obesity has increased over the past 20 years, and is associated with urbanization, low level of education, and older age, and often coincides with a history of T2D, hypertension, and/or cardiovascular disease [70]. However, there is also a micronutrient deficiency in the population, and this double burden of nutritional disorders is encountered by many developing countries [71,72]. Similarly, the United Arab Emirates and Mexico have also experienced a rapid increase in obesity and T2D and have responded by introducing sugar taxes and taking other measures.

Despite strategic interventions, planning and policies, governments have failed to meet the nutritional challenges of society. A significant rise in NCDs has been observed, mainly resulting from nutrition and obesity. In addition, there is micronutrient deficiency in selected societies and populations, which can increase the burden of disease. One of the main reasons for this problem is that nutrition is not a priority for policy makers, possibly due to the following reasons:

- Consequences of poor nutrition are often not immediately obvious and may manifest years later.
- Many people with malnutrition belong to poor and financially weak levels of society.
- Most policy makers have a clinical view of nutrition and less attention is paid to prevention and the social aspects of nutrition [71,73].
- Sometimes a conflict of interest exists between industry, governments and the health of the population [74].
- Indirect costs are not always apparent.
- Nutrition research is often biased (funded by industry) [74] and contradictory.
- Solutions are not easy to identify and are multifactorial.

Although dealing with obesity may be a daunting prospect, the situation will likely only deteriorate further if we do not take action as a community. We have identified numerous appropriate responses that have had a lesser or greater beneficial impact on the food environment/food consumption, and we need to collate these approaches into a comprehensive program. The message needs to be consistent [49], but difficulties often arise when there are conflicts of interest [74]. Greater transparency is required between nutrition related industry (and government partnerships, if relevant) and the public, and conflicts of interest need to be acknowledged. Despite these conflicts of interest, choices need to be made that will benefit the health and nutritional status of the community. Obesity is a communal problem that has come about due to societal changes, and hence requires an equally comprehensive response.

- What might an adequate response look like?
- Introduce government policies (where necessary) to regulate the marketing of food products and ensure it is appropriate. This could include regulations on trans-fats, sugar and salt content of foods, as well as eliminating multi-buy specials on ultra-processed and junk foods.
- Health professionals to play a proactive role in policy-making [21].
- Ensure that foods are appropriately labelled such that the label is legible and informative for a person who has received little education.
- Workshops with community groups and nutritional/health/behavioral specialists to discuss the problem of poor nutrition and possible solutions. Involvement of the community to implement and support behavioral change.
- Promotion of culturally appropriate role models.
- Ban advertising of sugar sweetened beverages and eliminate sponsorship of events where promotion of unhealthy foods is part of the contract.
- Introduce a sugar tax to discourage the purchase of sugar sweetened products.
- Remove tax from unprocessed, fresh foods.
- Provide a subsidy for fresh fruit and vegetables, if necessary, to ensure they are more affordable than energy dense snacks and junk foods.
- Remove advertising of ultra-processed and junk foods from in and around schools, and from children’s TV.
- Ban promotion of junk food to children (no rewards provided).
- No fundraising within schools where ultra-processed, energy dense foods are sold or won.
- Education and fun activities promoting healthy nutrition in schools. These should also include the local community.
- Activities to engage parents/caregivers through the schools, so that the changes at school are encouraged in the home.
- Workshops for caterers, working in school, factory and office cafeterias, aimed at improving the nutritional content of the meals they provide, and minimizing sugar, salt and fat content.
- Junk food outlets not permitted within a certain distance of schools, within or near hospitals and universities.
- Online educational resources providing culturally appropriate guidelines and cooking demonstrations available to the general public.
- Wide spread marketing to promote the consumption of unprocessed legumes, fruit and vegetables.
- Continual assessment of policies/interventions implemented, and adjustments made as necessary.
- Nutrition research to advance the response to obesity and poor nutrition.

From studying interventions to prevent and minimize malnutrition in young children, Nisbett et al. concluded that it may be easier to work with underlying and basic determinants of the problem (e.g., improving the infrastructure to enable easier access to education), rather than work from the top down by starting with the immediate determinants of health and nutrition (e.g., dietary intake) [75]. We suggest, when tacking excess calorie intake and unhealthy diets, the above-mentioned approach will very much depend on the resources and infrastructure within each local community. However, the basic determinants should be considered and addressed first if necessary.

It should be acknowledged that high fat and high sugar foods can be addictive [76,77], and therefore obesity and poor-nutrition may be much more challenging to resolve than malnutrition. Basically, we need to change our relationship with food if we are to avoid a future with a health care system over-burdened with nutritionally related NCDs, and a situation where the life expectancy of today’s youth is less than it is now. Furthermore, to avoid this situation we need to act now by providing the necessary support to parents/caregivers to feed their infants appropriately and avoid the development of poor nutritional habits and obesity. 

The discussion in this report reflects the effective implementation of a number of interventions to improve community health. Some of the above mentioned approaches, such as establishing surveillance systems, ensuring that guidelines are widely disseminated, execution and implementation of research (findings), and expansion of effective public and community health programs, are classic tools by which public and community health has addressed the burden of health and medical conditions for many years. However, expansion of this approach is required. The cautious use of the legislature and regulatory systems, by influencing consumer behavior through indirect-taxation or by shaping (and re-shaping) it through monitoring action, has become a progressively important intervention in contemporary community health practice in many of the interventions described above.

## 6. Conclusions

It is acknowledged that evidence, based on statistics on rising obesity, (or simply observing whilst walking through a number of cities distributed globally), shows that we are not doing enough to combat obesity and its inevitable contribution to NCDs. Obesity is a global problem, and it is not only a problem for the individual; collectively we need to create an environment that is supportive of the consumption of healthy foods, and appropriate levels of physical activity. However, in spite of the lack of a “one-size-fits-all” solution, it is clear that a comprehensive approach involving global and government programs, schools, and the community, is essential with respect to improving nutrition and decreasing the prevalence of obesity. The impact of obesity and poor nutrition on health is indisputable, as is the influence of marketing on the consumer. Government policies are required to eliminate the marketing of poor quality, energy dense foods, much like what has been implemented with tobacco use. Sin taxes should be widely expanded or introduced, and used to fund nutritious, unprocessed foods such that they are affordable for everyone. Community based support is required to ensure education, skills and motivational activities are implemented locally to enable and embed the required changes in lifestyle. If we wish to avoid unnecessary human suffering and avoid the reversal of the advances made in health care and economics, we need to acknowledge that a comprehensive, government supported plan is required to tackle the epidemic of obesity.

## Figures and Tables

**Table 1:** Dietary outcomes of nutritional intervention studies that took place in schools since 2000. Findings are presented in chronological order, with the most recent publications listed first.

Citation	Name of Study/Location	Study Design	Main Outcomes
[44]	Shaping Healthy Choices (Sacremento, USA)	Controlled trial (it was not clear if the trial was randomized)	In intervention schools, the prevalence of overweight and obese children decreased from 56% to 38% in one year.
[41]	New South Wales, Australia	RCT	Student purchases were lower in total fat but not calories or sodium.
[45]	SNAK (Michigan, USA)	Quasi-experimental intervention (online self-assessment)	Self-reported increased intake of fruit and fiber, and decreased intake of cholesterol.
[46]	HealthKick (Western Cape, South Africa)	RCT (reported by the school)	Some nutrition related activities were implemented in schools (e.g., availability of fruit in tuck shops), but on the whole the response was poor.
[47]	Rural Northern India	Cluster RCT (self-reported questionnaire)	Increase in fruit and vegetable consumption, decrease in consumption of deep-fried foods; no change in salty snack consumption
[48]	Discovering Health Choices (California, USA)	Intervention – Grade 4 students (unknown if RCT)	Measurable increase in student nutrition knowledge
[43]	HEALTHY (USA)	RCT	Lowered fat content of foods served; increased fiber content of foods served in the breakfast program; eliminated sugar sweetened beverages from the lunch program.
[42]	New South Wales, Australia	Quasi-experimental intervention (reported by school Principals)	The implementation of fruit and vegetable breaks increased to a greater extent in intervention schools.
[35]	CATCH (BPC vs BP) (Texas, USA)	Serial cross-sectional design (BP and BPC interventions)	Decreased intake of unhealthy foods in BPC schools. No difference in healthy food index score.
[49]	Maine, USA	Observational study pre and post implementation of State guidelines (survey)	Decrease in availability of soda. Pervasive availability of other sugar sweetened beverages and junk food. Advertising of “banned” foods in the school environment.
[50]	Maine, USA	Non-randomized quasi-experimental, prospective study.	Increased availability of low-fat, low-sugar and portion-controlled foods in schools.
[51]	TEENS (Minnesota, USA)	RCT	Intervention schools offered a higher proportion of healthy foods than control schools. No change in fruit and vegetable sales was observed.
[52]	Cafeteria Power Plus (Minnesota, USA)	RCT (observation)	Increase in fruit consumption
[53]	TACOS	RCT (environmental intervention)	Higher percentage of sales of low-fat foods in years 1 and 2 of the intervention. No self-reported change.
[54]	Pathways (American-Indian school children, USA)	Intervention study with control (it is unclear whether schools were randomized)	Mean reduction in total fat intake was observed in intervention schools from baseline to study end, and no change was observed in control schools.
[55]	El Paso CATCH (USA/Mexico border region)	20 intervention and 4 control schools	Decreased fat in school meals. Decreased sodium in school breakfasts but not lunches.
[56]	5-a-Day Power Plus (Minnesota, USA)	RCT (surveys and observation)	Improvement in school lunch intake (increased intake of fruit and vegetables, vitamin C and calcium, and decreased percentage calories from fat)

Abbreviations: BP – BasicPlus; BPC – BasicPlus Community; CATCH – Coordinated Approach To Child Health; RCT – Randomised Controlled Trial (schools were randomised, not students); SNAK – School Nutrition Advances Kid; TACOS – Trying Alternative Cafeteria Options in Schools; TEENS – Teens Eating for Energy and Nutrition at School.

**Table 2:** Anthropometric outcomes of nutritional intervention studies that took place in schools since 2000. Findings are presented in chronological order, with the most recent publications listed first.

Citation	Name of Study	Study Design	Main Outcomes
[57]	SNaX (5 week) (Los Angeles, USA)	RCT	Decrease in BMI in obese children in both intervention and control groups after two years (greater decrease in intervention group).
[58]	HEALTHY (USA)	Cluster RCT	Decrease in BMI; decrease in percentage of students with waist circumference ≥90^th^ percentile in both intervention and control schools.
[35]	CATCH (BPC vs BP) (Texas, USA)	Serial cross-sectional design (BP and BCP interventions)	Greater reduction in proportion of overweight and obese students in the BPC (8.3% decrease) vs BP (1.3% decrease) schools.
[54]	Pathways (American-Indian school children, USA)	Intervention study with control (it is unclear whether schools were randomized)	No changes were observed in body composition.
[55]	El Paso CATCH (USA/Mexico border region)	20 intervention and 4 control schools	No difference in weight or waist-to-hip ratio. Rate of increase was significantly less in the intervention schools.

Abbreviations: BP – BasicPlus; BPC – BasicPlus Community; CATCH – Coordinated Approach To Child Health; RCT – Randomised Controlled Trial (schools, not students, were randomised), SNaX – Students for Nutrition and Exercise.
